# Level Up Your Residency: Can a Novel Two-Week Simulation Rotation Really Make a Difference?

**DOI:** 10.7759/cureus.64569

**Published:** 2024-07-15

**Authors:** Lyndsey N Nguygen, Thomas C Hartmann, Doug W Harrington, Brian R Pulford

**Affiliations:** 1 Diagnostic Radiology, Naples Comprehensive Health (NCH) Healthcare System, Naples, USA; 2 Pulmonary Critical Care, Naples Comprehensive Health (NCH) Healthcare System, Naples, USA; 3 Internal Medicine, Naples Comprehensive Health (NCH) Healthcare System, Naples, USA

**Keywords:** interprofessional collaboration, medical education, transitional year residency, curriculum development, experiential learning, simulation in medical education

## Abstract

Introduction: This study describes a unique two-week simulation-based medical education (SBME) rotation for transitional year (TY) residents. During the rotation, residents are fully integrated into the simulation team, actively participating in clinically based interprofessional scenarios, procedural techniques, and mixed reality experiences. Residents also created and ran their own simulations while receiving content expert feedback. We evaluated the rotation's effectiveness in preparing TY graduates for their specific advanced residency track.

Methods: A retrospective survey evaluated the experiences of 11 TY residents who participated in a unique two-week simulation rotation. The survey assessed residents' perceptions of the program's value and skill development, course design, scenario relevance to future practice, and preparedness to develop future scenarios.

Results: Residents (11 out of 12 residents, 92% response rate) overwhelmingly endorsed the simulation rotation (100% positive, 45.45% extremely valuable). The program demonstrably improved core clinical skills (100% reported improvement) and fostered self-efficacy for future practice. Scenario relevance was high (81.82% highly relevant). Collaboration and communication skills showed promise (72.73% positive) while highlighting a potential area for future refinement. Residents unanimously agreed on effective time allocation and the program's value for debriefing skills. Notably, 91% strongly supported residency-specific simulation training.

Discussion: The two-week simulation was perceived by prior TY residents as valuable, with a majority finding the experience highly valuable across multiple survey questions. Residents overwhelmingly expressed a preference for residency-specific training, suggesting future development of specialty-tailored modules and enhanced debriefing sessions. The findings highlight the program's effectiveness and successful implementation into a TY residency curriculum.

## Introduction

Simulation medicine is an underutilized yet extremely useful tool in medical education, whether at the undergraduate or graduate medical education level [1]. Not every residency program in the United States offers such an opportunity to participate in simulation-based medical education (SBME). A Comprehensive Health Multidisciplinary Simulation Center, in conjunction with the Transitional Year (TY) Residency Program in the Graduate Medical Education (GME) Department, developed a two-week simulation rotation unique to TY resident physicians. Their experience also provided interprofessional support for SBME. Resident physicians were responsible for planning and developing a unique self-created scenario, dry-running their scenario, running their scenario, and receiving formal feedback from an attending physician fellowship-trained in simulation medicine [2]. Successful completion of the rotation is one of the core requirements of the TY Residency Program. The two-week experience incorporated the formal goals and objectives of the rotation, which included the expectation of a completed, authored scenario at the end of the two weeks. Goals and objectives for the rotation were set in accordance with the TY Milestones as established by the Accreditation Council for Graduate Medical Education (ACGME), namely (1) patient care, (2) medical knowledge, (3) systems-based practice, (4) practice-based learning and improvement, (5) professionalism, and (6) interpersonal and communication skills [3]. The primary goal of this study was to query previous TY resident physicians who have successfully completed the two-week simulation rotation and graduated from the TY Residency Program to assess the utility of the rotation and how this prepared them for their future categorical residencies in various specialties, such as radiology, dermatology, and physiatry.

## Materials and methods

Expectations and design of the two-week simulation rotation

The TY residents were integrated into the simulation team during their two weeks. The specific educational goals and objectives are based on the ACGME core competencies [3]. The expectations for the TY resident included being exposed to a wide range of simulation activities across different disciplines. This included but was not limited to Advanced Cardiovascular Life Support (ACLS) training, point-of-care ultrasound (POCUS), Stop the Bleed®, procedural techniques, and a mixed-reality immersion room experience, for example. Resident physicians were also responsible for planning and developing a unique self-created scenario, dry-running their scenario, running their scenario, and receiving formal feedback from an attending physician fellowship trained in simulation medicine. The TY resident had required readings including orientation to the medical simulation environment, conceptual frameworks in medical simulation, mastery learning and simulation, designing a simulation scenario, psychological safety, debriefing theories and methods, moulage, standardized patients, and simulation training and skills assessment. At the beginning of each week, an operations meeting occurred with an agenda that included the simulation calendar review for the next two weeks of activities, scenarios, task training, learners, courses, and classes that will be occurring for review and preparation. The TY resident worked with each key member of the simulation team, including the operation specialist/technician, the medical director, and the simulation coordinator. The TY resident was encouraged to attend aspects of simulation activities that would relate to the TY residency and their future specialty training. They were expected to attend a wide range of sessions to broaden their exposure to and understanding of the application of simulation in education, including planning meetings, scenario development, dry runs, and debriefing. At the completion of the two-week rotation, an evaluation was sent via the MedHub evaluation system for the resident to assess the rotation.

Study design and participants

This study employed a descriptive, retrospective design to evaluate the experiences of TY residents who participated in a newly developed two-week simulation rotation (established in 2021) in a Comprehensive Health Multidisciplinary Simulation Center. The rotation aimed to provide TY residents with broad exposure to all aspects of SBME. The target population comprised all 12 TY residents who graduated from the TY Residency Program during the years 2021-2023 after completing the two-week simulation rotation and are currently enrolled in an advanced residency program. The demographics of the TY residents included 10 radiologists, one dermatologist, and one physicist. This study was approved by the Institutional Review Board of Naples Comprehensive Health (approval number: IRB0051) prior to data collection.

Data collection

An electronic survey instrument was developed and deployed using SurveyMonkey (SurveyMonkey, San Mateo, CA, USA) to assess the residents' perspectives on the two-week simulation rotation. Questions were created and designed by the paper’s authors, as seen in the Appendices section. An initial pool of survey questions was developed using the research objectives as a guide. Through careful review by the authors, questions were selected that best aligned with the paper’s objectives while ensuring clarity and comprehensiveness. A Likert scale was used to capture diverse perspectives and to help maintain data consistency. The inclusion criteria for the survey included TY residents who completed a two-week simulation medicine rotation. Exclusion criteria included TY residents who did not complete the simulation rotation during the residency tenure. The survey was sent to residents after they completed their TY year and during enrollment in their advanced residency program. A Likert scale was used for all questions, with response options ranging from 0 to 5 (1 = strongly disagree, 5 = strongly agree). The survey focused on three key areas: first, residents' perceptions of their involvement in the rotation, including opportunities for learning and skill-based development in SBME; second, residents' evaluation of the overall value and perceived benefits of the two-week simulation rotation; and third, residents' insights on how the acquired simulation skills could be applied to their future clinical practice.

The survey was sent to the 12 target residents through an email invitation. All residents who completed the two-week simulation rotation were invited to participate in the study. Participation was voluntary and anonymous. Of the 12 TY graduates, 11 (92%) responded and completed the survey.

Descriptive statistics were used to summarize the quantitative data collected through the SurveyMonkey questionnaire. Due to the small sample size (n=11), non-parametric statistics (median) were used for central tendency alongside percentages for all questions. The data was analyzed thematically to identify recurring patterns and key concepts in the residents' experiences.

## Results

Residents overwhelmingly endorsed the value of the two-week simulation rotation for their TY training, as seen in Table 1. All respondents (n=11) reported positive experiences, with five (45.45%) finding it extremely valuable. This suggests the program effectively addressed core SBME learning objectives for TY residents.

**Table 1:** Survey results for the two-week simulation rotation

Question	5 (highest)	4	3	2	1 (lowest)
Value of rotation	5	6			
Improved skills	7	4			
Scenario relevance	8	1	2		
Improved preparedness	7	4			
Collaboration and communication	3	6	2		
Course structure	8	3			
Debriefing skills	7	4			
Support for simulation integration	10		1		

Based on the Likert scale responses, all residents reported improvement in problem-solving, critical thinking, and decision-making skills, with seven (63.64%) strongly agreeing. This aligns with their perception of the program's value, suggesting the rotation fostered both theoretical understanding and practical application of these crucial skills.

Self-authored scenarios by the TY residents emerged as a key strength. Nine (81.82%) residents found the simulation scenarios relevant to the challenges faced in their post-TY advanced specialty training, with eight (72.73%) expressing high relevance. This highlights the program's success in incorporating resident experiences into the curriculum, potentially leading to increased engagement and perceived value.

Improved preparedness was another notable outcome. All 11 respondents felt more prepared to handle cases and develop future scenarios after completing the program. Notably, seven (63.64%) strongly agreed. This suggests a positive shift in residents' self-efficacy, which is crucial for a successful transition to advanced training.

Collaboration and communication skills emerged as another strength of the program, with eight (72.73%) residents agreeing it enhanced their ability to work effectively within the simulation environment. This finding highlights the program's success in fostering teamwork through its collaborative scenarios and debriefing sessions focused on communication dynamics within the healthcare team. While two (27.27%) residents provided neutral responses, this offers an opportunity to explore areas for further refinement and maximize the program's impact. Future research could utilize open-ended questionnaires to pinpoint areas where the program can be tailored to better address individual needs.

Effective time allocation and debriefing skills received unanimous praise. All residents agreed on the program's success in allocating sufficient time for scenario development and experiential learning. All residents also found the program provided valuable foundations for debriefing and communicating clinical events encountered during advanced specialty training. These findings highlight the importance of both elements in SBME and suggest that the program effectively addressed these critical aspects.

**Figure 1:**
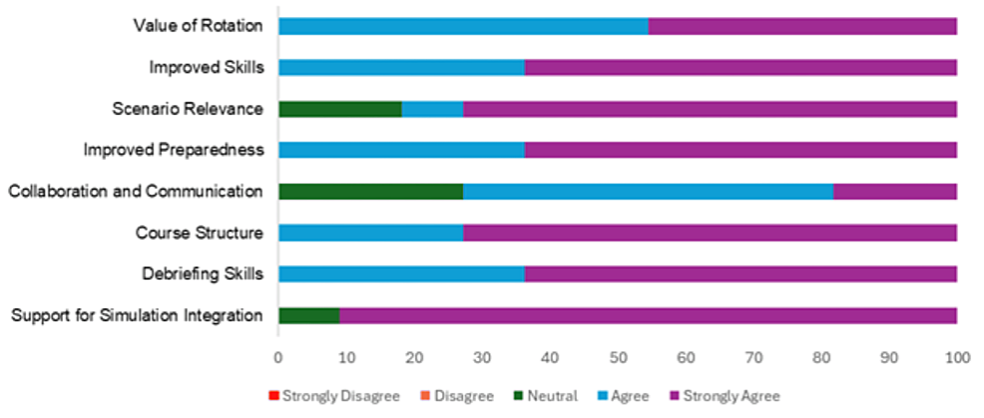
Residents' feedback on the two-week TY simulation program TY: transitional year

**Figure 2:**
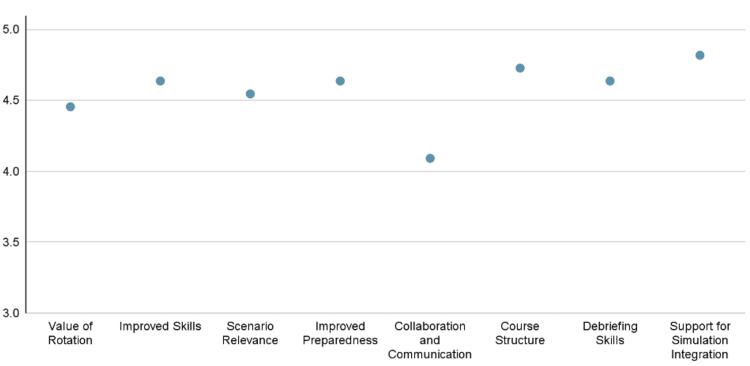
Mean participant response to individual survey questions This graph depicts the mean Likert scale scores (1 = strongly disagree, 5 = strongly agree) across various survey topics (X-axis). It reveals participants' mean level of agreement with statements related to each topic.

The residents overwhelmingly supported the concept of residency-specific simulation training, as illustrated in Figures 1-2. Ten (91%) residents strongly agreed on the value of tailoring future programs to specific residencies. This finding highlights a potential area for future program development with the potential for a high impact on resident preparedness for their chosen specialty.

## Discussion

This study investigated the experiences of TY residents participating in a newly developed two-week simulation rotation program in the Comprehensive Health Multidisciplinary Simulation Center. The findings provide valuable insights into the program's effectiveness and areas for potential improvement.

Perceptions of program value and core skill development

The two-week simulation rotation proved immensely valuable for TY training, with all responding residents perceiving it as beneficial and a significant portion finding it extremely valuable. The survey results suggested a positive impact on core skills crucial for advanced specialty training, such as problem-solving, critical thinking, and decision-making. These findings demonstrate the program's potential to enhance residents' preparedness for future success in their chosen specialties [4]. These findings are consistent with numerous studies demonstrating the effectiveness of simulation in enhancing these essential skills for healthcare professionals [5].

Scenario relevance and resident preparedness

**Figure 3:**
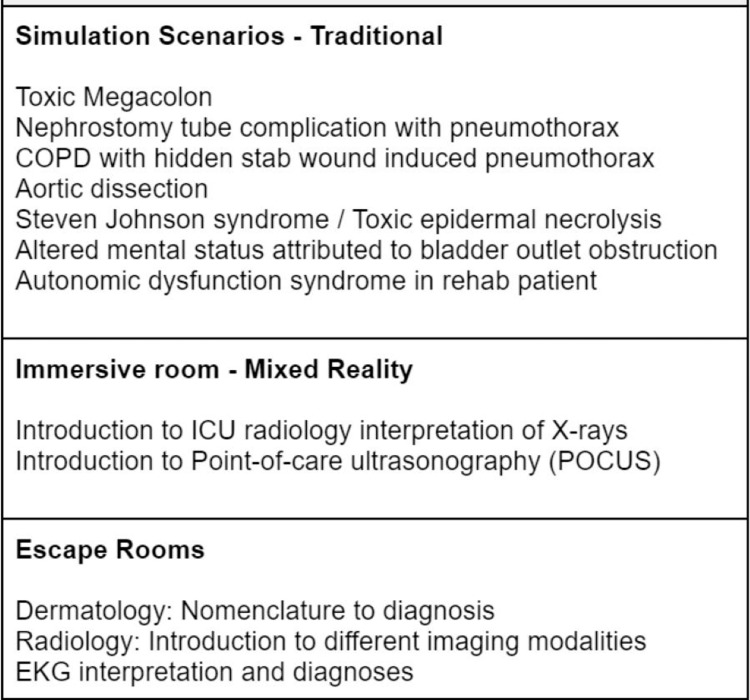
Self-authored TY simulation scenarios TY: transitional year, COPD: chronic obstructive pulmonary disease, ICU: intensive care unit, EKG: electrocardiogram

Encouragingly, the majority of residents found the self-authored simulation scenarios relevant to the challenges they faced in their advanced specialty training. This suggests the program effectively bridges the gap between theory and practical application, enhancing resident preparedness. The self-authored scenarios created by TY residents during their simulation rotation can be seen in Figure 3. To further refine the rotation and ensure alignment with future TY resident needs, it could be beneficial to reach out to program directors from these advanced specialties (i.e., dermatology, radiology, physiatry, etc.) to elicit additional recommendations on simulation scenarios faced by residents in these fields.

Collaboration and communication skills

The program demonstrably improved collaboration and communication with support staff (nurses, technicians, educators, etc.). A significant portion of residents agreed that the simulation rotation enhanced these crucial skills. This suggests the program's design, incorporating interprofessional scenarios and teamwork-focused debriefing sessions, fosters effective collaboration within the healthcare team, an important aspect of any residency training.

While some residents provided neutral responses, their experiences hold valuable insights for further program development. Future research with a larger sample size could utilize open-ended interviews to explore these experiences in more detail. This deeper understanding could help tailor the program's scenarios and debriefing sessions to address any communication or collaboration challenges residents encounter while working with support staff within the simulation environment.

Course design and content

This study emphasizes the critical role of well-structured course content and design in maximizing the educational benefits of short-term SBME programs. All responding residents perceived the program as aligned with its goals and reported a deeper understanding of SBME principles, highlighting the effectiveness of the curriculum.

Furthermore, residents agreed the program provided a valuable foundation for debriefing and communication skills, essential for effective SBME implementation. This strongly suggests the program's dedicated debriefing sessions, designed to facilitate discussions around case analysis, application, and reflective learning, had a positive impact [6]. These findings underscore the importance of structured course design and content in maximizing the educational value of a two-week simulation-based learning experience.

Resident preferences and future program development

Residents overwhelmingly favored the incorporation of advanced residency-specific simulation training, reflecting the current trend in healthcare education toward personalized learning experiences [7,8]. To address this preference, our program could explore developing residency-specific modules and experiences dedicated to specific residencies with content experts [9]. To ensure scenario and learning objective relevance, these experiences would target the unique challenges residents face in their chosen specialties. This could involve using specialty-specific case examples or focusing on skills particularly relevant to advanced residency tracks. The resident and content expert’s input would remain crucial in optimizing scenario development and maximizing the educational benefit. By implementing these strategies, our program can effectively cater to resident preferences for a more personalized learning experience while still benefiting from the core structure and existing resources. This approach ensures the program's adaptability, addresses the specific needs of TY residents across various specialties, and ultimately maximizes its effectiveness in preparing them for their chosen career paths.

Limitations

This study, while offering valuable insights, has limitations. The small sample size (n=11) necessitates further research with a larger resident population for generalizable findings. Furthermore, the study relied primarily on self-reported data from surveys. Future research with a larger and more diverse resident population is necessary to confirm these findings. Additionally, incorporating objective assessments of resident performance before and after the program could offer a more robust evaluation of its impact on skill development. Finally, although we cannot assess the generalizability of our findings to other educational programs, the methods utilized, combined with open-ended interviews, could be applied in future evaluations of the effectiveness of a non-traditional two-week simulation rotation.

## Conclusions

This study successfully implemented a novel two-week simulation rotation for TY residents. Residents overwhelmingly endorsed the program's value, with a significant portion finding it extremely valuable, underscoring its effectiveness. Descriptive analyses illustrated that TY residents developed improvements in their confidence in core problem-solving, critical thinking, and decision-making skills and felt more prepared for advanced training. Notably, the high relevance of self-authored simulation scenarios further strengthened the program's impact.

These findings contribute to the growing body of evidence supporting the effectiveness of SBME in residency programs. This study particularly highlights the promise of resident involvement in learner-centered scenario development. Scenarios tailored to their specific needs and interests not only enhance engagement but also create a more relevant learning experience. Additionally, the unanimous resident support for effective debriefing practices underscores the critical role of SBME in residency training.

## Appendices

Survey questions

1. How valuable did you find the two-week simulation rotation in enhancing your transitional year training?

5 = Extremely valuable

4 = Valuable

3 = Neutral

2 = Somewhat valuable

1 = Not valuable

2. The simulation rotation improved my approach to problem-solving, critical thinking, and decision-making experienced during my transitional year residency.

5 = Strongly agree

4 = Agree

3 = Neutral

2 = Disagree

1 = Strongly disagree

3. Were the developed/authored simulation scenarios relevant to the challenges faced by resident in their post-transitional year advanced specialty training?

5 = Highly relevant

4 = Relevant

3 = Neutral

2 = Somewhat relevant

1 = Not relevant

4. I feel more prepared to handle cases and develop future scenarios after completing this simulation rotation.

5 = Strongly agree

4 = Agree

3 = Neutral

2 = Disagree

1 = Strongly disagree

5. The simulation rotation enhanced my collaboration and communication skills among support staff (i.e., nurses, technicians) during my advanced specialty training.

5 = Strongly agree

4 = Agree

3 = Neutral

2 = Disagree

1 = Strongly disagree

6. The simulation course effectively allocated time for understanding the basics of simulation scenario development and experiential learning.

5 = Highly effective

4 = Effective

3 = Neutral

2 = Somewhat effective

1 = Not effective

7. The simulation rotation provided the foundations for debriefing and communicating clinical events that are encountered during my advanced specialty training.

5 = Strongly agree

4 = Agree

3 = Neutral

2 = Disagree

1 = Strongly disagree

8. Do you think residency-specific simulation training would be beneficial in your present residency?

5 = Strongly agree

4 = Agree

3 = Neutral

2 = Disagree

1 = Strongly disagree
